# Assessing the impact of COVID-19 lockdown on quality of life: a cross-sectional study of university staff in Borneo

**DOI:** 10.7717/peerj.21179

**Published:** 2026-06-09

**Authors:** Boon Tat Yeap, M. Tanveer Hossain Parash, May Zaw Soe, Sadia Choudhury Shimmi

**Affiliations:** 1 Department of Anesthesiology and Intensive Care, Faculty of Medicine and Health Sciences, Universiti Malaysia Sabah, Kota Kinabalu, Sabah, Malaysia; 2 Department of Biomedical Sciences, Faculty of Medicine and Health Sciences, Universiti Malaysia Sabah, Kota Kinabalu, Sabah, Malaysia; 3 Department of Medical Education, Faculty of Medicine and Health Sciences, Universiti Malaysia Sabah, Kota Kinabalu, Sabah, Malaysia

**Keywords:** COVID-19, Lockdown, Quality of life, WHOQOL-BREF, Borneo

## Abstract

**Background:**

The COVID-19 pandemic and its associated lockdown measures have drastically impacted daily life, particularly among vulnerable populations. University staff faced significant challenges during the COVID-19 lockdown, particularly after restrictions were imposed, requiring them to quickly adapt to e-learning and remote work. Given the limited research on this topic and the intriguing findings of previous studies, this research seeks to address the gap by exploring the distinct challenges faced by university staff and offering strategies for improved support during future disruptions. This study investigates the quality of life (QoL) of both academic and non-academic staff at the university during the Movement Control Order (MCO), with a focus on identifying the factors that influence QoL.

**Methods:**

This cross-sectional study investigates the QoL among academic and non-academic staff at the university during the first MCO. Data were collected *via* an online survey conducted from May 2020 to April 2021, utilizing the WHOQOL-BREF questionnaire to evaluate physical health, psychological well-being, social relationships, and environmental domains.

**Results:**

Among 427 participants, significant demographic insights emerged: over two-thirds were female, one-third held diplomas, and the majority faced financial constraints. QoL scores varied significantly across domains, with the widest distribution in the environmental domain. No gender-based differences in QoL scores were observed, but educational attainment, duration of employment, marital status, and family income significantly influenced QoL outcomes. Participants with higher education levels or longer employment durations generally reported better QoL. Married participants and families with higher incomes or fewer children also exhibited better scores in social and environmental domains. These findings underscore the profound interplay between socioeconomic and demographic factors in shaping QoL during lockdowns. The results highlight the urgent need for targeted interventions addressing financial stress and mental health challenges to mitigate the negative impacts of prolonged restrictions on vulnerable groups.

## Introduction

COVID-19 has significantly changed our way of life since its onset in December 2019. The virus soon widened its spread across the globe and was declared a pandemic by WHO on March 11, 2020 (World Health, 2020). The first record of Malaysia’s COVID-19 cases was recorded on January 25, 2020, and as of March 31, 2020, the reported cases had increased drastically to 2,766 positive cases with 43 deaths within two months (Elengoe, 2020). As a countermeasure, the Malaysian government implemented several non-pharmaceutical measures to curb the spread of COVID-19, including different phases and levels of movement restrictions such as the Movement Control Order (MCO), Conditional MCO, and Recovery MCO (Zamri et al., 2021). The MCO, introduced on March 18, 2020, was the strictest phase of Malaysia’s COVID-19 measures, allowing only essential economic activities and banning interstate travel and gatherings (Shah et al., 2020; Aziz et al., 2020; Murphy et al., 2023). These measures effectively curbed the spread of COVID-19 and flattened the epidemic curve during Malaysia’s second wave, as reported by Gill et al. (2020) and Aziz et al. (2020). Similar events were reported worldwide by other researchers (Murphy et al., 2023; Andronico et al., 2021; Lau et al., 2020; Atalan, 2020).

Lockdown measures affected almost every aspect of daily life. Positive effects such as improved air and water quality were reported due to reduced industrial activity (Kanniah et al., 2020). Digital adoption accelerated, with remote work, online education, and e-commerce becoming essential (Abu Talib, Yunus & Mazlan, 2021). Families also reported increased bonding time, as the restrictions allowed individuals to reconnect with loved ones and focus on home-based activities, contributing to stronger familial ties (Tan, Cheah & Lee, 2021). However, negative consequences were also widespread, including financial strain, job insecurity, social isolation, and increased psychological distress (Kumar & Nayar, 2020; Labrague, De Los Santos & Falguera, 2021; Ferreira et al., 2021; Zhang & Ma, 2020).

The COVID-19 lockdown not only affected occupational and financial aspects of participants’ lives but also significantly altered lifestyle behaviors, which may have contributed to quality-of-life outcomes. Many studies have reported disruptions in sleep patterns, irregular eating habits, and reductions in physical activity during periods of confinement (Hargreaves et al., 2021; Li et al., 2022). These behavioral changes were often accompanied by increased sedentary time and screen exposure, potentially leading to weight gain and exacerbating psychological stress (Msherghi et al., 2021; Prithviraj et al., 2023). Moreover, lockdowns were associated with heightened engagement in addictive behaviors, including tobacco, alcohol, and other substance use, as coping mechanisms for stress and social isolation (Kumar & Nayar, 2020; Zhang & Ma, 2020). The shift to remote work also imposed additional household burdens, such as balancing work, childcare, and domestic responsibilities, which intensified feelings of fatigue and disrupted routines (Aye, Tan & Ramasamy, 2024; Tan, Cheah & Lee, 2021). Collectively, these lifestyle and behavioral disruptions may have compounded the psychological and environmental stressors observed in our study, contributing to domain-specific declines in quality of life.

During the COVID-19 lockdown, quality of life (QoL) was notably impacted, but the degree of this impact was not uniform and differed among groups from diverse backgrounds (Epifanio et al., 2021). Higher education staff faced abrupt shifts to remote teaching, administrative duties, and student support, often without adequate infrastructure or training (Ismail et al., 2021; Onyema et al., 2020; Coman et al., 2020). Balancing increased workloads with family responsibilities created further stress (D’Silva et al., 2021). Early studies documented reduced QoL among university staff during the pandemic (Onyema et al., 2020; Rahman et al., 2024), More recent research provides a more nuanced picture. Some studies show persistent stress and burnout among higher education employees (Brandau, Vogt & Garey, 2022), while others describe adaptive strategies and partial recovery of well-being (Anda & Tay, 2024; Aye, Tan & Ramasamy, 2024).

Despite growing research on the mental health and QoL impacts of COVID-19, most studies have focused on healthcare workers, students, or populations in urban centers. Very few investigations have targeted university staff, and even fewer have explored institutions in geographically distinct regions such as Borneo. The unique socioeconomic, infrastructural, and cultural contexts of universities in Borneo may influence staff experiences differently from those in peninsular Malaysia or other countries. Addressing this gap is critical for developing targeted policies and institutional support mechanisms that are sensitive to regional diversity.

This study aims to assess the QoL of both academic and non-academic staff in Borneo during the initial MCO period and identify demographic and socioeconomic factors influencing QoL. We hypothesized that (1) university staff would differ in QoL scores during the MCO, and (2) sociodemographic and pandemic-related factors would be significantly associated with QoL across its domains. Findings are intended to inform institutional strategies to better support staff during future public health emergencies.

## Materials & Methods

This cross-sectional questionnaire-based study was carried out during the period starting from May 2020 until April 2021.

### Inclusion criteria

Academic and non-academic staff of the university having official email address were included in the study.

### Exclusion criteria

Outsourced staff were excluded from the study.

### Study population

Using the formula *n* = Z^2^P(13P)/d^2^ (*Z* = 1.96, *P* = 0.5, *d* = 0.05), the required sample was 384; after finite population correction for *N* = 2,971, this was reduced to 341 (Kelsey et al., 1996).

### Instrumentation

The study utilized a structured, self-administered online questionnaire consisting of three sections:

(1) Sociodemographic data: Age, sex, marital status, education level, job category (academic or non-academic), household income, and number of dependents.

(2) Pandemic-related factors: Changes in work modality, workload, childcare responsibilities, and perceived institutional support.

(3) Quality of life assessment: QoL was measured using the World Health Organization Quality of Life-BREF (WHOQOL-BREF) instrument, a validated 26-item tool assessing physical health, psychological health, social relationships, and environment domains (The WHOQOL Group, 1998). Responses were rated on a 5-point Likert scale, and domain scores were transformed to a 03100 scale, with higher scores indicating better QoL.

### Data collection procedures

The survey was hosted on a secure institutional platform. Participants completed informed consent form and could complete the questionnaire in approximately 15 to 20 min. No personally identifying information was collected to ensure anonymity. Based on prior experience with the same population and anticipating a 50% likelihood of low participation, the questionnaire link was randomly distributed to 682 academicians and non-academicians at the university *via* official email addresses, accompanied by a request for participation. To maximize response rates, reminders were sent twice during the data collection period. If more than four items in the questionnaire were missing, the questionnaire was excluded from the study.

#### Ethical permission

The medical research ethics committee of the Faculty of Medicine and Health Sciences, Universiti Malaysia Sabah, provided the ethical approval (Ref no: UMS/FPSK6.9/100-6/1/95) for this research.

#### Data analysis

At first, the data were screened for normality, and the data were not normally distributed (Table 1). Hence, median, quartiles, interquartile range (IQR), and range were used to describe the data. To compare the different QoL domains between two categories Mann–Whitney *U* test and more than two categories Kruskal–Wallis *H* test were performed. *p*-value less than 0.05 was considered as the level of significance. All the statistical analysis was performed using SPSS Software Version 27.0.

**Table 1:** Tests of normality.

	**Kolmogorov–Smirnov** ^a^			**Shapiro–Wilk**		
	**Statistic**	**df**	**Sig.**	**Statistic**	**df**	**Sig.**
Physical domain	0.072	427	0.000^*^	0.991	427	0.010^*^
Psychological domain	0.120	427	0.000^*^	0.973	427	0.000^*^
Social domain	0.155	427	0.000^*^	0.938	427	0.000^*^
Environmental domain	0.065	427	0.000^*^	0.987	427	0.001^*^

**Notes.**

a Lilliefors significance correction.

* Significant at *p* < 0.05 level.

## Results

Among the four hundrd and twenty-seven participants, more than two-thirds were female and held a permanent position in the university (Table 2). One-third had diplomas, one-fourth had bachelor’s degrees, and one-fifth held master’s degrees or higher. Nearly half had served the university for fewer than five years, while one-fourth had worked at least ten years. The majority were single and childless, with about half reporting family incomes below RM 4,849; most did not experience financial constraints during the MCO.

**Table 2:** Demographic profile of the participants (*n* = 427).

		**Frequency**	**Percent**
Gender	Male	106	24.8
	Female	321	75.2
Educational qualification	SPM	34	8.0
	STPM/Matriculation	42	9.8
	Diploma	138	32.3
	Bachelor	115	26.9
	Masters	49	11.5
	PhD	49	11.5
Status of Position	Contract	98	23.0
	Permanent	329	77.0
Duration of service at UMS	<1 year	78	18.3
	1–5 years	191	44.7
	6–10 years	44	10.3
	>10 years	114	26.7
Marital status	Single	260	60.9
	Divorced	3	0.7
	Married	164	38.4
Number of children	Nil	276	64.6
	1–3	113	26.5
	4–6	35	8.2
	7 and more	3	0.7
Combined family income per month	<RM 4,849	230	53.9
	RM4,850 - RM10,959	141	33.0
	≥ RM 10,960	56	13.1
The presence of financial constraints during thisMCO	No	271	63.5
	Yes	156	36.5
	Total	427	100.0

Table 3 depicts the median, quartiles, interquartile range, and range of the participants. Median QoL scores varied across domains, with the widest distribution observed in the environmental domain and the narrowest in the psychological domain.

**Table 3:** Descriptive statistics of different domains among the participants (*n* = 427).

**Domains**	**Range**	**1**st **percentile**	**Median**	**3**rd **percentile**	**IQR**
Physical domain	6.29–18.86	10.86	12.57	13.71	2.86
Psychological domain	6.00–18.00	11.33	12.00	13.33	2.00
Social domain	6.67–17.33	12.00	14.67	16.00	4.00
Environmental domain	5.50–20.00	12.50	14.50	16.50	4.00

**Figure 1:**
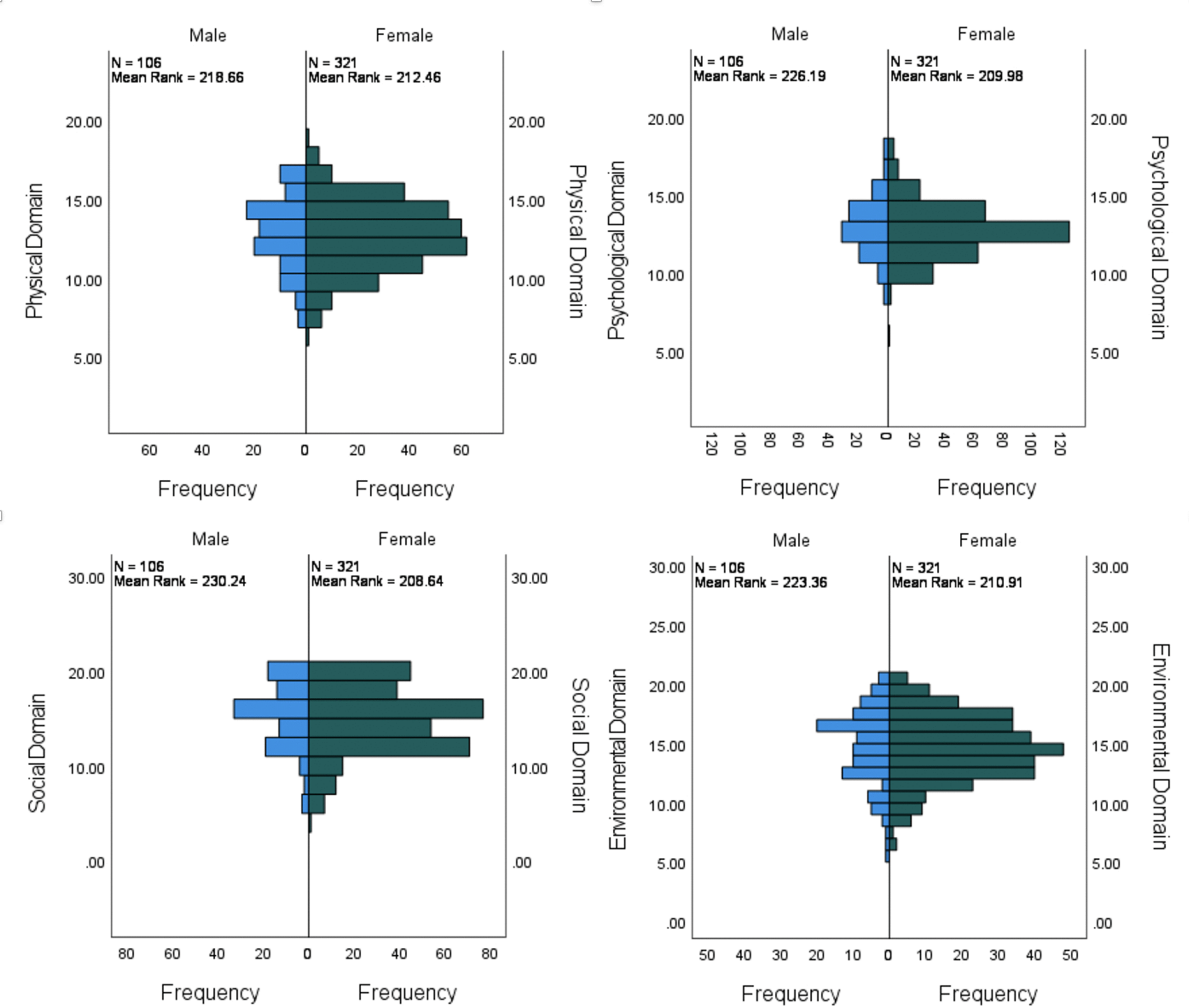
Frequency of WHOQOL scores across the gender of the participants (n-427).

**Figure 2:**
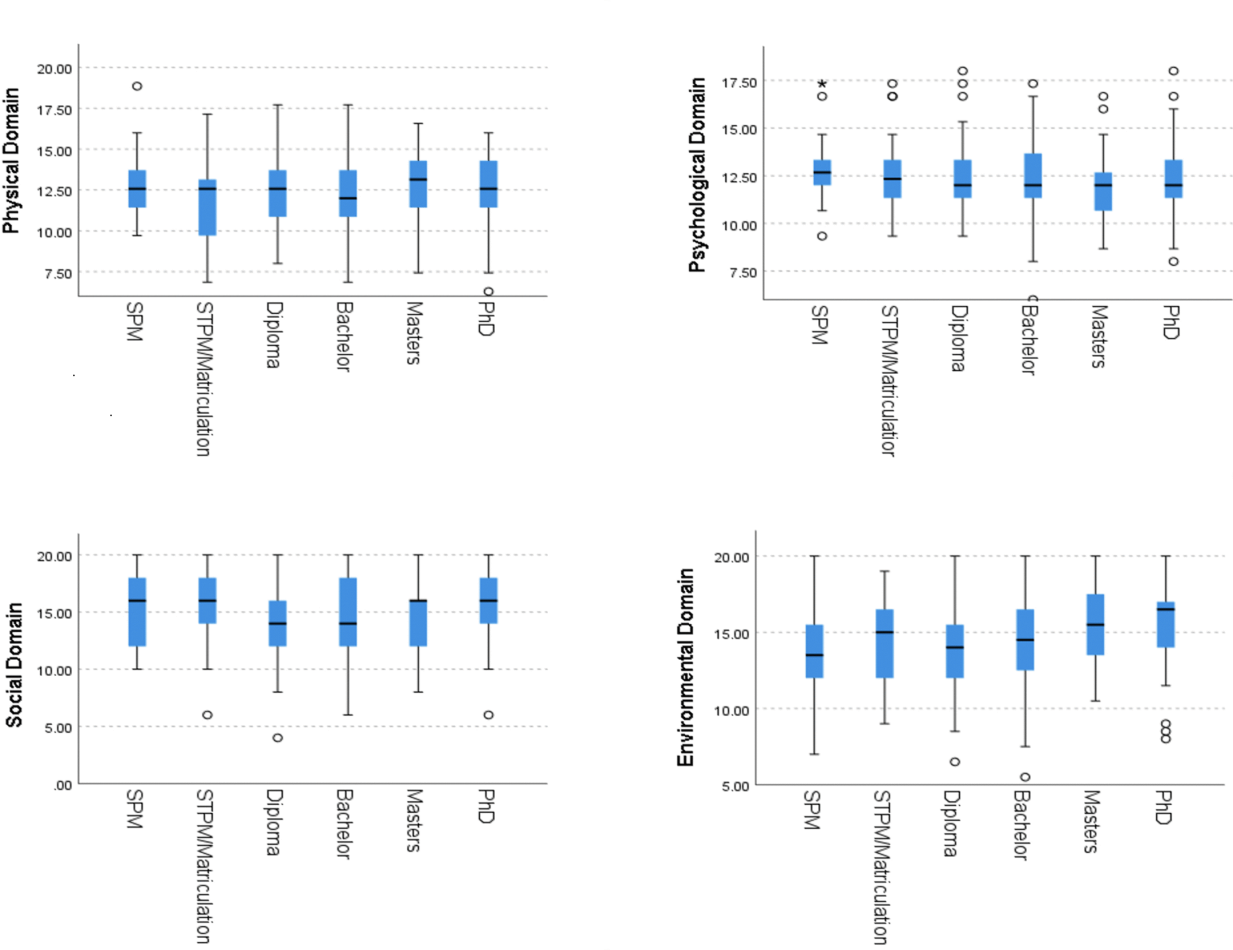
Range and median of WHOQOL scores across the educational qualification (n-427).

**Figure 3:**
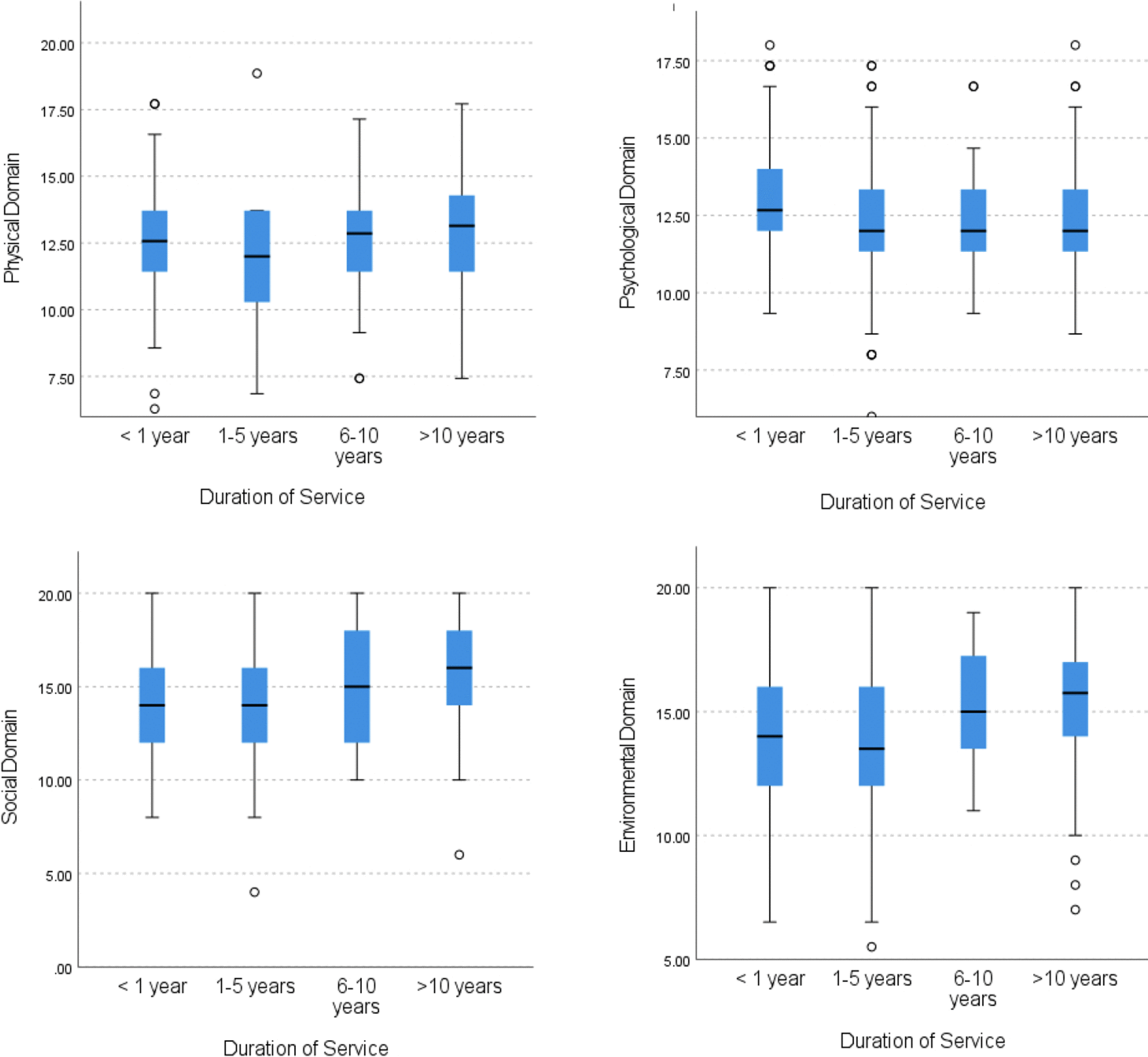
Range and median of WHOQOL scores across the duration of service (n-427).

**Figure 4:**
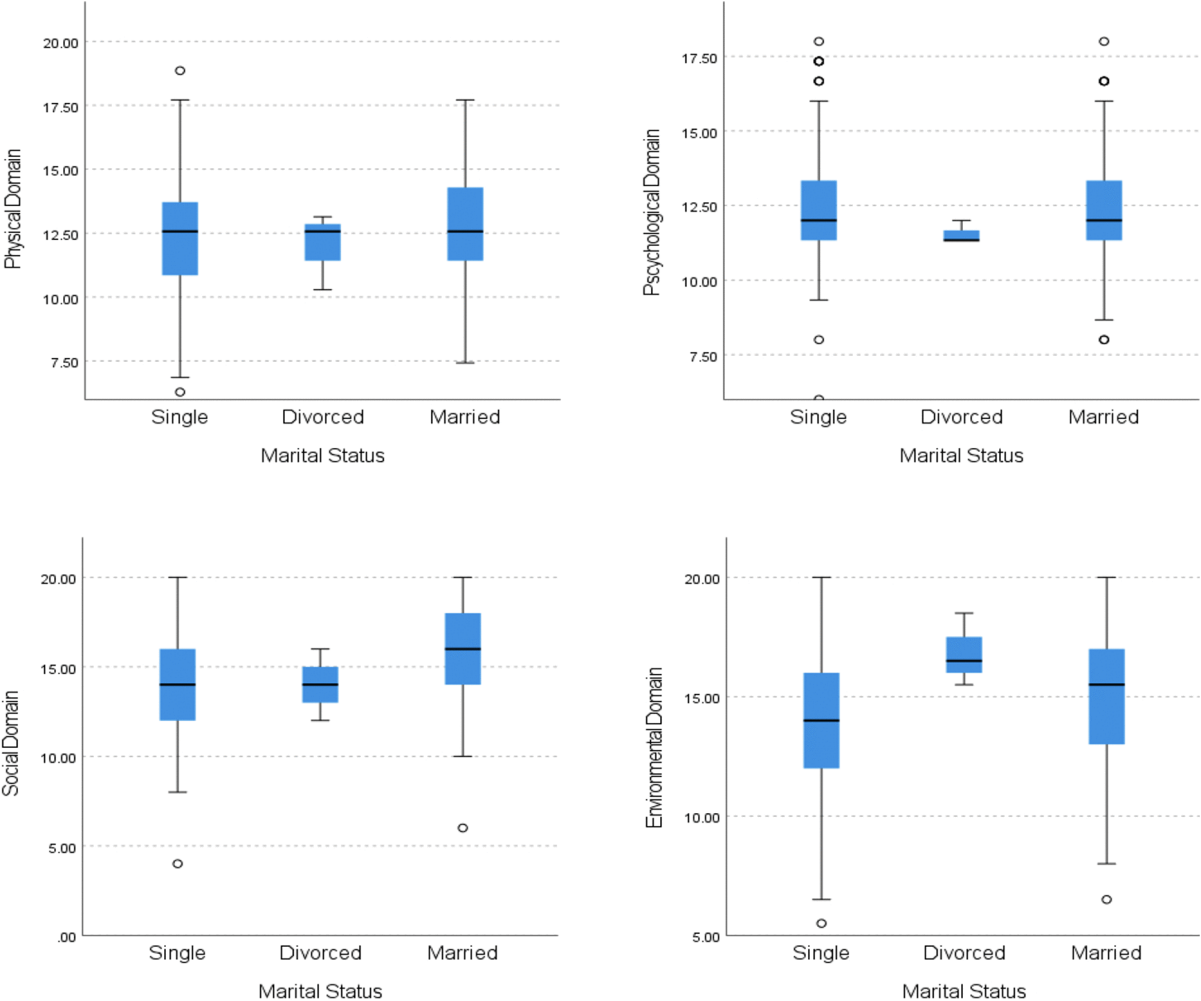
Range and median of WHOQOL scores across the marital status of the participants (n-427).

There was no significant difference in QoL scores between males and females across all domains (*p* > 0.05; Fig. 1, Table 4). However, participants with PhDs reported higher environmental QoL scores compared to diploma holders (*p* = 0.001; Fig. 2, Tables 5 and 6). The QoL scores was significantly different in both social and environmental domains when the participants were distributed in different duration of service (Fig. 3 and Table 7). The highest difference in social and environmental domains was between those working for five years and those working at least for ten years (*p* < 0.001; Table 8). In the environmental domain, those working for less than one year were significantly lower than those working for at least ten years (*p* < 0.001; Table 8). The married scored higher than the single participants both in social and environmental domains (*p* < 0.001; Fig. 4, Table 9, and Table 10).

**Figure 5:**
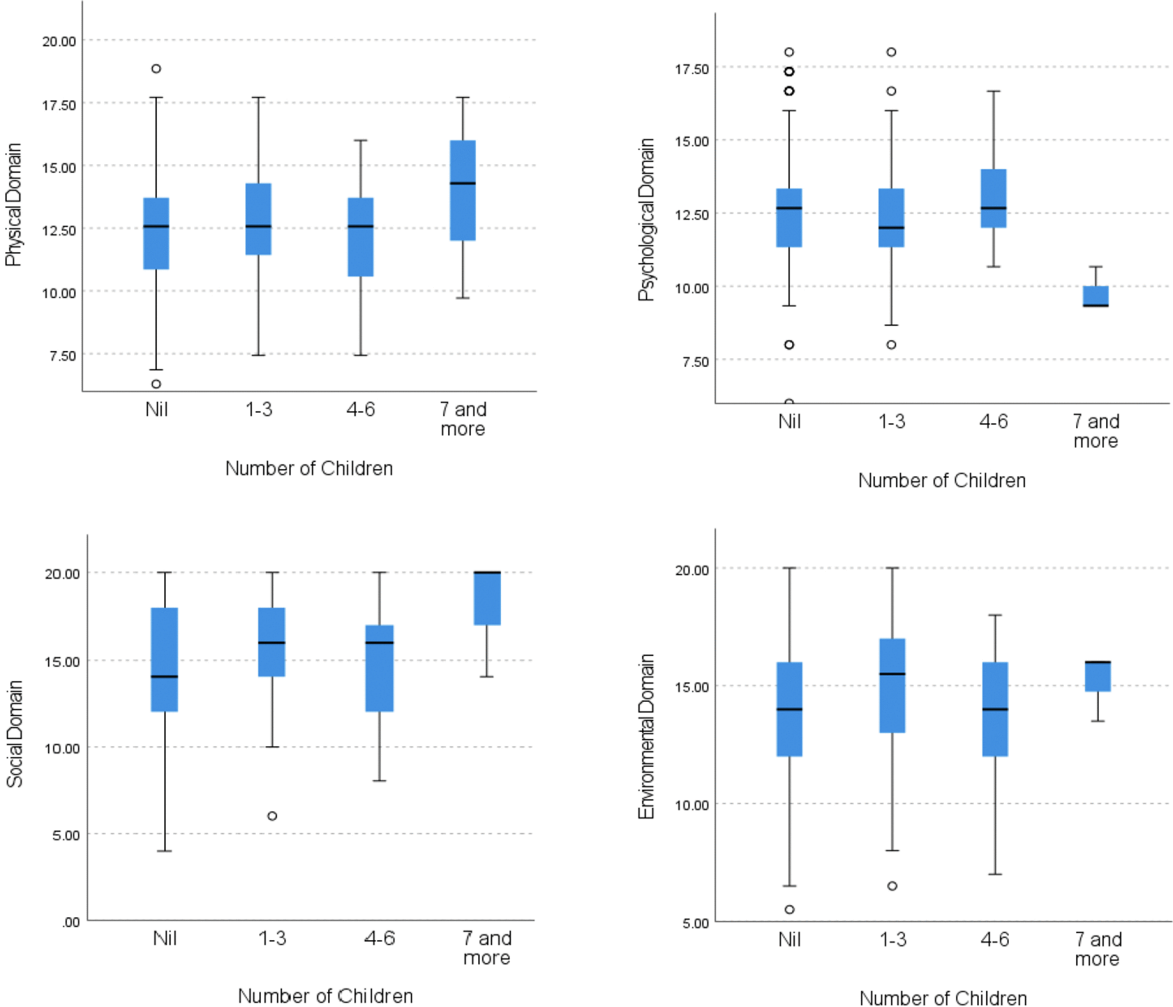
Range and median of WHOQOL scores across the number of children in the family (n-427).

**Figure 6:**
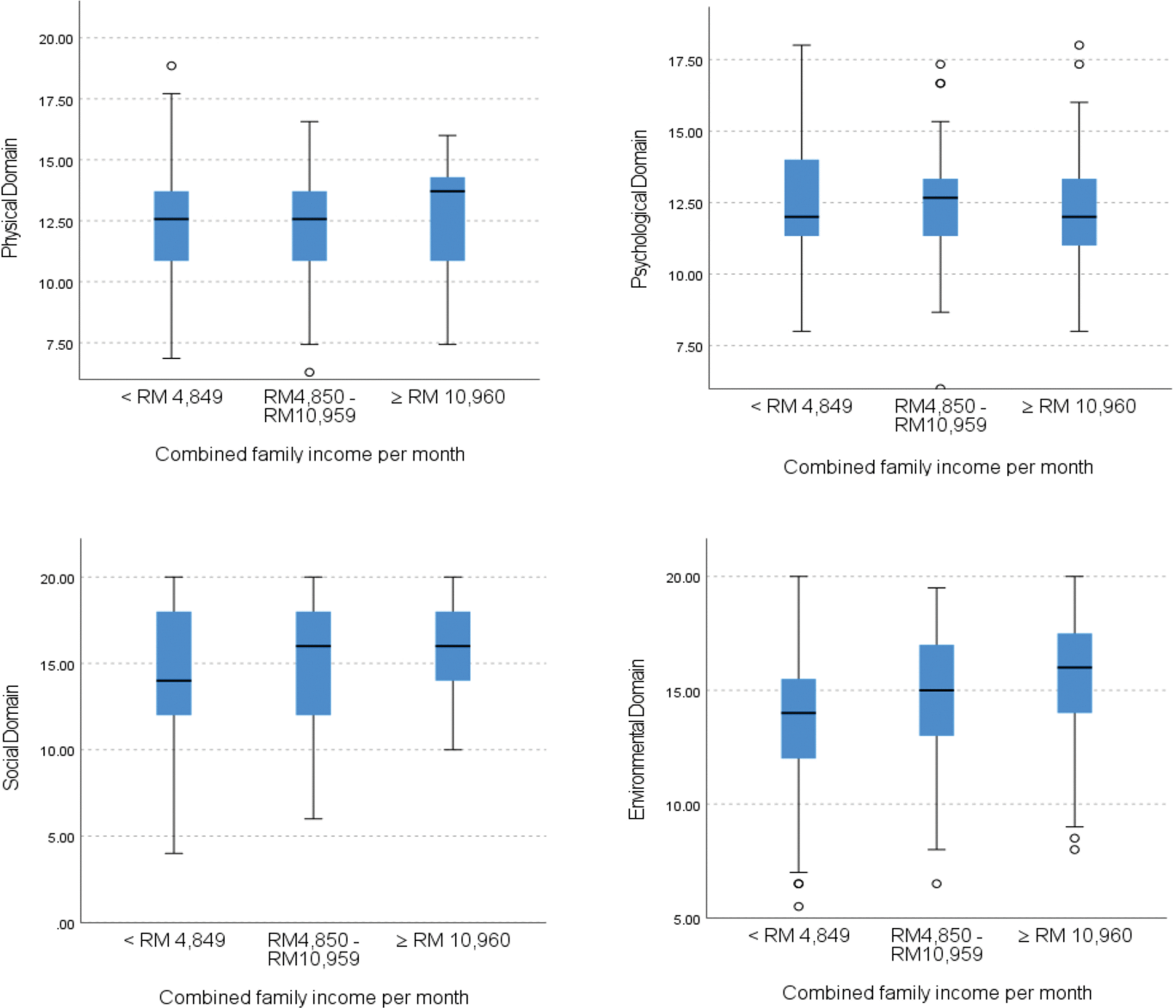
Range and median of WHOQOL scores across the combined family income per month (n-427).

Families with at least seven children had lower QoL score in the psychological domain than families with four to six children, one to three children, and no child (*p* < 0.017; Fig. 5, Table 11, and Table 12). Families with one to three children had significant low score than families without a child in social and environmental domains (*p* = 0.013 and *p* = 0.037; Table 12). The social and environmental domain scores were significantly higher (*p* < 0.001) for the families who had combined income per month of at least RM 10,960 than those having the same less than RM 4,849 (Fig. 6, Table 13, and Table 14). The families with a combined family income less than RM 4,849 also differed in the social domain from those with the same between RM4,850 and RM10,959 (Table 14).

**Figure 7:**
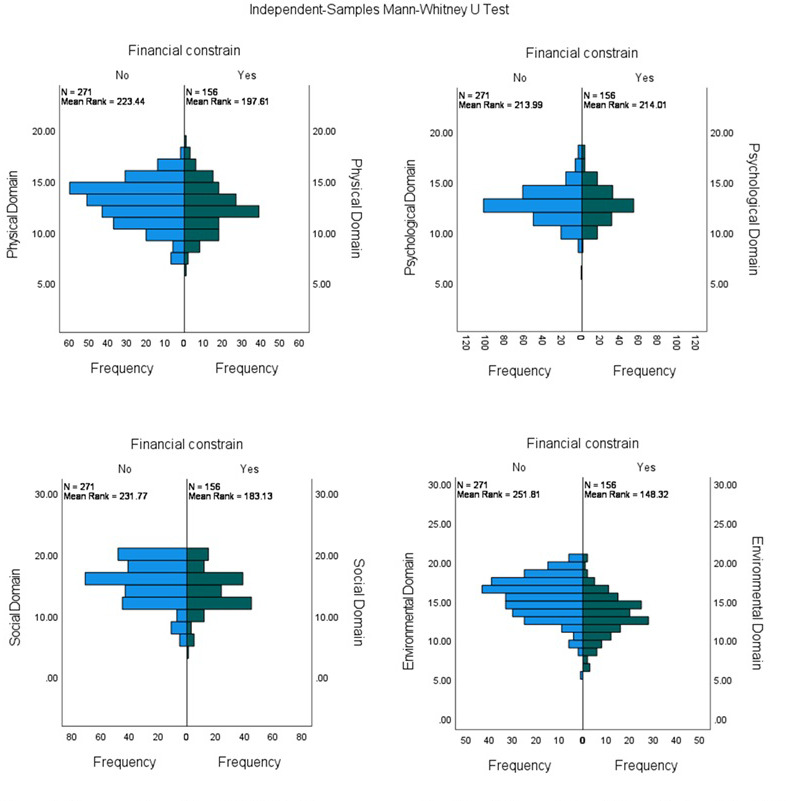
Range and median of WHOQOL scores across the financial constrain of the participants (n-427).

Financial constraint was also decisive in physical, psychological, and environmental domain scores (Fig. 7). Those having financial constrain had highly significant low score (*p* < 0.001) in the environmental and psychological domain and mildly significant low score (*p* = 0.037) in the physical domain (Table 15).

## Discussion

The aim of this study was to examine local perceptions and responses to the implementation of the MCO lockdown and to explore the factors influencing quality of life (QoL) among university staff. Drawing on data from 427 participants, the findings provide important insights into the demographic and socioeconomic patterns shaping QoL during the early phase of the COVID-19 pandemic. The sample was predominantly female, highly educated, and largely composed of individuals holding permanent university positions. Educational attainment ranging from diplomas to postgraduate degrees served as an important indicator of socioeconomic resilience, a trend also highlighted in previous pandemic-related research (Smith et al., 2022; Msherghi et al., 2021). Higher education is closely associated with greater job security, digital literacy, and access to resources that may buffer individuals from the negative impacts of lockdown measures

**Table 4:** Comparison of WHOQOL scores based on the gender of the participants (*n* = 427).

	**Mann–Whitney U**	**Standard error**	**z**	**Asymp. sig.**
Physical domain	16,519.50	1,098.125	−0.449	0.653^ns^
Psychological domain	15,721.00	1,091.03	−1.184	0.236^ns^
Social domain	15,291.50	1,081.86	−1.591	0.112^ns^
Environmental domain	16,020.50	1,099.90	−0.902	0.367^ns^

**Notes.**

ns Non-significant at *p* < 0.05 level.

**Table 5:** Comparison of WHOQOL scores based on educational qualification (*n* = 427).

	**Kruskal–Wallis H**	**df**	**Asymp. sig.**
Physical domain	4.916	5	0.421^ns^
Psychological domain	4.313	5	0.505^ns^
Social domain	9.797	5	0.081^ns^
Environmental domain	23.672	5	0.000^*^

**Notes.**

ns Non-significant at *p* < 0.05 level.

* Significant at *p* < 0.001 level.

**Table 6:** Pairwise comparisons of environmental domain across educational qualification (*n* = 427).

**Sample 1–sample 2**	**Test statistic**	**Std. error**	**z**	**Sig.**	**Adj. sig.** ^a^
SPM-PhD	−84.181	27.502	−3.061	0.002	0.033^*^
Diploma-Masters	−67.865	20.490	−3.312	0.001	0.014^*^
Diploma-PhD	−80.437	20.490	−3.926	0.000	0.001^*^
STPM/Matriculation-PhD	−61.298	25.910	−2.366	0.018	0.270
Bachelor-PhD	−48.961	21.020	−2.329	0.020	0.298

**Notes.**

Each row tests the null hypothesis that the Sample 1 and Sample 2 distributions are the same.

* Asymptotic significances (2-sided tests) are displayed. The significance level is *p* < .050.

a Significance values have been adjusted by the Bonferroni correction for multiple tests.

**Table 7:** Comparison of WHOQOL scores based on the duration of service (*n* = 427).

	**Kruskal–Wallis H**	**df**	**Asymp. sig.**
Physical domain	9.968	3	0.019^*^
Psychological domain	5.071	3	0.167^ns^
Social domain	17.864	3	0.000^*^
Environmental domain	27.252	3	0.000^*^

**Notes.**

ns Non-significant at *p* < 0.05 level.

* Significant at *p* < 0.001 level.

**Table 8:** Pairwise comparisons of physical, social, and environmental domain across the duration of service (*n* = 427).

	**Sample 1-sample 2**	**Test statistic**	**Std. error**	**z**	**Sig.**	**Adj. sig.** ^a^
Physical domain	1–5 years->10 years	−43.919	14.559	−3.017	0.003	0.015^*^
Social domain	1–5 years->10 years	−58.559	14.344	−4.083	0.000	0.000^*^
	<1 year->10 years	−52.996	17.809	−2.976	0.003	0.018^*^
	6–10 years->10 years	−45.101	21.509	−2.097	0.036	0.216
Environmental domain	1–5 years-6–10 years	−58.407	20.604	−2.835	0.005	0.028^*^
	1–5 years->10 years	−67.953	14.583	−4.660	0.000	0.000^*^
	<1 year-6–10 years	−54.571	23.231	−2.349	0.019	0.113
	<1 year->10 years	−64.118	18.106	−3.541	0.000	0.002^*^

**Notes.**

Each row tests the null hypothesis that the Sample 1 and Sample 2 distributions are the same.

* Asymptotic significances (2-sided tests) are displayed. The significance level is *p* < .050.

a Significance values have been adjusted by the Bonferroni correction for multiple tests.

Financial challenges emerged as a notable issue, with nearly half of the participants reporting family incomes below RM 4,849. This finding reflects the broader economic strain associated with lockdown measures, consistent with evidence showing that lower-income groups experienced heightened vulnerability during the pandemic (Rahaman et al., 2021; Smith et al., 2022). International studies similarly indicate that lockdowns disproportionately affected individuals in lower-paying jobs and younger age groups, contributing to increased financial instability and psychological distress (Li et al., 2022; Prithviraj et al., 2023). These patterns underscore the strong linkage between economic strain and reduced QoL during periods of restricted mobility and social disruption.

Across QoL domains, the environmental domain displayed the greatest variability, suggesting that external conditions such as living environment, access to services, and financial security were central to participants, experiences during the lockdown. These findings parallel results from WHOQOL-based studies conducted during the pandemic. Homayuni et al. (2022) and Triantafillou et al. (2024) both reported that environmental and psychological domains were especially sensitive to pandemic-related stressors, including confinement, limited mobility, and reduced access to public services. Similarly, Singh et al. (2021) observed declines in environmental QoL among healthcare workers, driven by safety concerns and increased work demands. Although our sample was not composed of frontline workers, the observed patterns indicate that environmental stressors remained salient across occupational groups.

Notably, no gender differences in QoL were detected, aligning with studies suggesting that gender does not universally predict psychological responses to lockdown measures (Hargreaves et al., 2021). However, educational level was a significant determinant of environmental QoL, with PhD holders reporting higher scores than diploma holders. This supports previous evidence linking higher education to enhanced coping capacity, better access to resources, and more stable working conditions during crisis periods (Msherghi et al., 2021). Duration of service also contributed meaningfully to QoL outcomes: individuals with longer employment tenures reported better social and environmental QoL than those with fewer years of service. Longer-tenured staff may benefit from greater job security, institutional familiarity, and established support networks, which can mitigate the negative impacts of lockdown (Smith et al., 2022).

**Table 9:** Comparison of WHOQOL scores based on marital status (*n* = 427).

	**Kruskal–Wallis H**	**df**	**Asymp. sig.**
Physical domain	3.868	2	0.145^ns^
Psychological domain	1.738	2	0.419^ns^
Social domain	16.919	2	0.000^*^
Environmental domain	13.454	2	0.001^*^

**Notes.**

ns Non-significant at *p* < 0.05 level.

* Significant at *p* < 0.001 level.

**Table 10:** Pairwise comparisons of physical, social and environmental domain across the marital status of the participants (*n* = 427).

	**Sample 1–sample 2**	**Test statistic**	**Std. error**	**z**	**Sig.**	**Adj. sig.** ^a^
Social domain	Single-married	−49.157	12.085	−4.068	0.000	0.000^*^
Environmental domain	Single-married	−40.478	12.287	−3.294	0.001	0.003^*^

**Notes.**

Each row tests the null hypothesis that the Sample 1 and Sample 2 distributions are the same.

* Asymptotic significances (2-sided tests) are displayed. The significance level is *p* < .050.

a Significance values have been adjusted by the Bonferroni correction for multiple tests.

**Table 11:** Comparison of WHOQOL scores based on number of children in the family (*n* = 427).

	**Kruskal–Wallis H**	**df**	**Asymp. sig.**
Physical domain	2.777	3	0.427^ns^
Psychological domain	10.136	3	0.017^*^
Social domain	10.710	3	0.013^*^
Environmental domain	8.508	3	0.037^*^

**Notes.**

ns Non-significant at *p* < 0.05 level.

* Significant at *p* < 0.001 levelstatus of the participants (*n* = 427).

**Table 12:** Pairwise comparisons of physical, social, and environmental domain across numbers of children in the family (*n* = 427).

	**Sample 1–sample 2**	**Test statistic**	**Std. error**	**z**	**Sig.**	**Adj. sig.** ^a^
Psychological domain	7 and more-1-3	170.131	71.495	2.380	0.017	0.104
	7 and more-Nil	188.768	70.947	2.661	0.008	0.047
	7 and more-4-6	208.981	73.526	2.842	0.004	0.027
Social Domain	Nil-1-3	−38.705	13.535	−2.860	0.004	0.025
Environmental domain	Nil-1-3	−37.430	13.761	−2.720	0.007	0.039

**Notes.**

Each row tests the null hypothesis that the Sample 1 and Sample 2 distributions are the same.

* Asymptotic significances (2-sided tests) are displayed. The significance level is *p* < .050.

a Significance values have been adjusted by the Bonferroni correction for multiple tests.

Marital status and family structure played significant roles in shaping QoL. Married participants generally reported higher QoL, particularly in the social and environmental domains, underscoring the protective influence of family support systems during periods of crisis (Prithviraj et al., 2023). Participants with one to three children had lower social and environmental QoL compared to those without children, reflecting challenges in balancing childcare, home responsibilities, and work demands during the lockdown. Families with large numbers of children exhibited significantly lower psychological QoL, consistent with studies documenting increased parenting stress and emotional burden during pandemic restrictions (Msherghi et al., 2021). Income-related disparities were also evident: families with lower combined incomes experienced marked reductions in QoL across multiple domains, reinforcing well-documented associations between financial strain and diminished psychological, social, and environmental well-being (Li et al., 2022).

However, the magnitude of impairment in the physical domain in the present study appears more modest compared with the psychological and social domains, a pattern that has also been reported elsewhere. Some studies among healthcare workers and academic staff found that while psychological distress increased substantially during lockdown, physical QoL scores were relatively preserved, possibly due to maintained employment, flexible working arrangements, or adaptive coping strategies (Pappa et al., 2020; Algahtani et al., 2021). In contrast, our findings highlight that financial constraints were significantly associated with lower physical QoL scores, suggesting that economic stress may exacerbate physical strain through mechanisms such as fatigue, reduced access to health-promoting resources, and disrupted daily routines. This underscores the importance of considering socioeconomic context when interpreting physical QoL outcomes, as financial insecurity may amplify the physical consequences of prolonged sedentary work and restricted movement. Overall, the present findings align with existing literature while also extending it by emphasizing that physical quality of life, although sometimes less visibly affected than mental health, remains a critical component of overall wellbeing during pandemic-related lockdowns.

It is important to recognize that the majority of respondents were embedded in a relatively stable employment sector that continued to function during the lockdown. Their experiences likely differ from individuals engaged in gig work, informal employment, or heavily impacted service industries. Workers in these vulnerable sectors faced greater job insecurity, more severe financial losses, and higher levels of stress, which may have contributed to substantially lower QoL than what is reflected in our sample. This distinction highlights the need for caution when generalizing the findings beyond relatively secure, salaried populations.

**Table 13:** Comparison of WHOQOL scores based on combined family income per month (*n* = 427).

	**Kruskal–Wallis H**	**df**	**Asymp. sig.**
Physical domain	2.222	2	0.329^ns^
Psychological domain	2.600	2	0.273^ns^
Social domain	6.639	2	0.036^*^
Environmental domain	24.680	2	0.000^*^

**Notes.**

ns Non-significant at *p* < 0.05 level.

* Significant at *p* < 0.001 level.

**Table 14:** Pairwise comparisons of physical, social, and environmental domain across combined family income per month (*n* = 427).

	**Sample 1–sample 2**	**Test statistic**	**Std. error**	**z**	**Sig.**	**Adj. sig.** ^a^
Social domain	<RM 4,849-≥ RM 10,960	−44.861	18.059	−2.484	0.013	0.039
Environmental Domain	<RM 4,849-RM4,850 - RM10,959	−45.613	13.179	−3.461	0.001	0.002
	<RM 4,849-≥ RM 10,960	−80.288	18.361	−4.373	0.000	0.000

**Notes.**

Each row tests the null hypothesis that the Sample 1 and Sample 2 distributions are the same.

* Asymptotic significances (2-sided tests) are displayed. The significance level is *p* < .050.

a Significance values have been adjusted by the Bonferroni correction for multiple tests.

(Conversion of 1 USD = MYR 4.47 as at November 2024).

**Table 15:** Comparison of WHOQOL scores based on financial constrain of the participants (*n* = 427).

	**Mann–Whitney U**	**z**	**Asymp. sig.**	**Adj. sig.** ^a^
Physical domain	18,581.00	−2.089	0.037^*^	0.021^*^
Psychological domain	21,136.50	−0.001	0.999^ns^	0.014^*^
Social domain	16,322.00	−3.994	0.000^*^	0.129^ns^
Environmental domain	10,892.00	−8.357	0.000^*^	0.000^*^

**Notes.**

a 1-tailed significance through Moses test where 13 outliers were trimmed from each end.

* Significant at *p* < 0.05 level.

ns Non-significant at *p* < 0.05 level.

While the dataset was robust and statistically consistent, a key limitation noted by the reviewer concerns the representativeness of the sample. Because complete institutional demographic records were not available, we were unable to compare the characteristics of respondents with the full university staff population. Thus, potential selection bias cannot be excluded. Individuals with more stable employment, higher digital access, or greater engagement with institutional communications may have been more likely to participate in the survey.

Additionally, although the WHOQOL-BREF is widely validated and extensively used in pandemic-related research (Homayuni et al., 2022; Rashid et al., 2024; Triantafillou et al., 2024), it may not fully capture pandemic-specific stressors such as remote-work fatigue, digital overload, household responsibilities, and sudden shifts in work-life boundaries.

These gaps may partly explain the domain-specific patterns observed. Further, the cross-sectional design prevents causal inference and cannot capture changes in QoL as the pandemic evolved. The absence of multivariate modelling also limits the ability to control for potential confounders, such as pre-existing mental health conditions or coping strategies. Future studies should consider longitudinal methods and incorporate robust or quantile regression approaches to identify independent predictors with greater precision.

Self-reported online questionnaires introduce additional challenges, including recall bias, socially desirable responding, and exclusion of individuals with limited digital access. These factors may have influenced patterns of participation and potentially underrepresent vulnerable subgroups.

Despite these limitations, the large sample size, aligned findings across QoL domains, and consistency with existing literature suggest that the results are meaningful and contribute valuable insight into the experiences of university staff during the MCO period.

## Conclusions

This study highlights the complex interplay between socioeconomic factors, education, family dynamics, and psychological well-being in shaping the impacts of lockdowns on quality of life. The findings underscore the need for targeted interventions to support vulnerable populations, particularly those facing financial instability or limited access to resources. These insights are invaluable for policymakers and mental health professionals as they work to address the challenges posed by the pandemic and its broader societal consequences.

## Supplemental Information

10.7717/peerj.21179/supp-1 Supplemental Information 1 Data

## Additional Information and Declarations

### Data Availability

The following information was supplied regarding data availability: The raw data are available in the Supplementary File.
